# Psychometric Properties of the Traditional Chinese Version of the Stanford Presenteeism Scale (SPS-6) among Taiwanese Employees in Technology Companies

**DOI:** 10.3390/healthcare10112202

**Published:** 2022-11-02

**Authors:** Kim-Ngan Ta-Thi, Kai-Jen Chuang

**Affiliations:** 1 School of Public Health, College of Public Health, Taipei Medical University, Taipei 11031, Taiwan; 2 Faculty of Public Health, University of Medicine and Pharmacy at Ho Chi Minh City, Ho Chi Minh City 700000, Vietnam; 3 Department of Public Health, School of Medicine, College of Medicine, Taipei Medical University, Taipei 11031, Taiwan

**Keywords:** presenteeism, validity, productivity, traditional Chinese, SPS-6, reliability, measurement properties, psychometric properties

## Abstract

Little attention has been paid by employers to reduced productivity at work due to illness (presenteeism) because valid instruments to measure presenteeism are lacking. We assessed psychometric properties of the traditional Chinese version of the six-item Stanford Presenteeism Scale (CSPS-6) among Taiwanese employees in technology companies. We carried out a cross-cultural adaptation study on 196 employees. Factor analyses were used to evaluate the construct validity of the CSPS-6. Cronbach’s alpha was 0.74. The content validity of the CSPS-6 was good. Results of factor analyses confirmed the two-factor model of the CSPS-6. CSPS-6 scores were correlated with job stress (r_s_ = −0.22, *p* = 0.002), the health status SF-36 (r_s_ = 0.28 to 0.52, *p* < 0.0001), job satisfaction (r_s_ = 0.41, *p* < 0.0001), and the presenteeism score of the Work Productivity and Activity Impairment Questionnaire: General Health (r_s_ = −0.46, *p* < 0.0001). No correlations were found between presenteeism and the disability status (*p* = 0.19, F-value = 1.67, degrees of freedom = 2). The CSPS-6 was found to be reliable and valid in evaluating presenteeism of Taiwanese employees. Further studies should be undertaken to validate the CSPS-6 in other working populations and assess long-term effects of health problems associated with presenteeism.

## 1. Introduction

Presenteeism is reduced productivity due to illness in employees who go to work but exhibit inadequate or unsatisfactory work performance [1,2]. Many employees still go to work when they are sick. This might affect their productivity and health because there is an association between sick leave and presenteeism [3].

Presenteeism is a key public health issue because it reduces work outcomes in employees and causes hidden indirect costs for organizations. In a cross-sectional study to estimate lost productive time caused by employees’ common pain conditions in the United States, presenteeism accounted for the majority (77%) of this lost productive time [4]. A study of all costs of health, absence, disability, and productivity losses for 10 disease conditions in US companies estimated that presenteeism costs accounted for the largest component, averaging 61%, of total costs for these diseases [5]. However, neither managers nor employees could define the extent of employees’ presenteeism. Only a few employers invested in the healthcare of their staff as they did not have information about indirect costs caused by their employees’ productivity loss due to health problems [1].

A comprehensive instrument to assess presenteeism is needed to evaluate the level of health-related productivity and prevent decreased productivity at work among employees. This means that decreased productivity at work might be considered an outcome to assess socioeconomic burdens of any health problems and evaluate impacts of health promoting interventions in the workplace or any healthcare system. These findings might contribute to health policies on whether or not managers and employers should invest in human capital [6].

To date, although many researchers have developed various instruments to measure presenteeism, there has been little consensus on how to measure presenteeism because different authors have different definitions of presenteeism [7]. Among 21 instruments measuring presenteeism in a systematic review in 2015, the Stanford Presenteeism Scale (SPS-6) had more evidence of its measurement properties than other instruments [8]. Until now, according to a systematic review in 2021, researchers have continued using it among 42 instruments of productivity loss in validation studies and economic evaluations [9]. Because the SPS-6 can possibly be used for all working populations and any health problems, it was translated and validated in several languages, such as Portuguese [10,11], Dutch [12], Italian [13], Persian [14], Spanish [15], and Turkish [16]. The developers of SPS-6 defined presenteeism as follows: “employees are physically present at their jobs, but they may experience decreased productivity and below-normal work quality”. Therefore, the SPS-6 has two factors: avoiding distractions and completing work which reflect work processes and work outcomes, respectively [6]. In previous validation studies, the SPS-6 was adapted into Italian [13], Portuguese [10,11,17], Dutch [12], Spanish [15], and Persian [14] with acceptable reliability (Cronbach’s alpha = 0.72~0.89) and construct validity of a two-factor model of the SPS-6. From these results of cross-cultural adaptation, we might generalize presenteeism or health-related productivity across countries.

Nevertheless, there are still limited validation data on this questionnaire. First, in Taiwan, the level of presenteeism or health-related productivity assessments of employees is not clear because valid instruments for its evaluation are still deficient. Hence, the main issue we addressed was to translate the SPS-6 into traditional Chinese (called the CSPS-6 version) and assess its psychometric properties. Second, because health problems are related to loss of productivity at work, SPS-6 scores were correlated with the health status (SF-36), disability status, job stress, and job satisfaction in previous validation studies [6,10]. In this study, we also hypothesized that the health status, disability status, job stress, and job satisfaction would be correlated with presenteeism as measured by the SPS-6 questionnaire among Taiwanese employees in technology companies.

## 2. Materials and Methods

Our methods included psychometric properties (or measurement properties), the process of translation, samples, measures, and data analysis.

### 2.1. Psychometric Properties

According to the classification of reliability and validity of health outcomes instruments in the COSMIN study [18], we evaluated the internal consistency, content validity, construct validity, and criterion validity. 

The test–retest reliability is not presented because the SPS-6 reflects the effects of health problems, such as flu and headaches, on the productivity in the previous month, and both health problems and productivity might change over time [6]. The content validity and translation equivalence were assessed by expert panels, the original developer of this scale, and two independent native English-speaking researchers.

The construct validity includes the structural validity and hypothesis testing [18]. To evaluate the structural validity of the CSPS-6, we performed an exploratory factorial analysis and a confirmatory factor analysis. For hypothesis testing, we hypothesized that the health status, disability status, job stress, and job satisfaction would be correlated with presenteeism on the basis of a literature review [6,10], because health problems are related to productivity losses at work.

Due to there being no gold standard instrument for assessing productivity loss [19], we evaluated the concurrent validity (a type of criterion validity) by comparing CSPS-6 scores with scores of a similar measure (the Work Productivity and Activity Instrument Questionnaire: General Health (WPAI:GH) presenteeism).

### 2.2. The Process of Translation

After gaining permission to validate the questionnaire from one of the original developers of the SPS-6, we followed Sousa and Rojjanasrirat’s guidelines of forward–backward translation [20]. Briefly, forward–backward translation was performed by two independent translators and synthesized by a third translator. Second, the original developer of this scale and two native English-speaking researchers worked independently to assess the content equivalence between the original version and the backward translation with four responses from 1 indicating “strongly disagree” to 4 indicating “strongly agree”. 

After that, we arranged two expert panels: one expert panel via email to evaluate the content validity and another face-to-face expert panel to finalize the translated version of the CSPS-6 (see Supplementary Materials). We conducted a pilot study with 12 employees to check the wording, and no changes were made to the final translated version.

### 2.3. Samples

According to Jackson [21] and Kline [22], the minimum sample size for a structural equation model for a confirmatory factor analysis (CFA) should be determined by the N:q ratio, with q being the number of parameters and N being the number of cases. Since the recommended N:q ratio is 20:1 and the SPS-6 has six parameters, the minimum sample size would be 20q or N = 120.

A cross-sectional design was used at 10 technology companies in the north of Taiwan. Inclusion criteria were Taiwanese employees, age of at least 20 years old, and ability to answer all questions of the CSPS-6. We recruited 196 employees by random sampling and interviewed them after obtaining informed consent.

### 2.4. Measures

Demographic data included age, gender, education, marital status, and occupational categories.

The RAND 36-item 1.0 questionnaire (also known as the SF-36 1.0) was utilized for measuring the health status. Eight subscales of the SF-36 are general health, physical functioning, pain, social functioning, vitality, role—emotional, role—physical, and mental health. Responses were recoded from 0 to 100 and averaged into scores of each subscale. Higher scores indicate better health [23].

Employees were asked about job satisfaction and job stress. Each response for job satisfaction or job stress was scored as follows: 1 = “completely dissatisfied” or “extremely low”, 2 = “moderately dissatisfied” or “low”, 3 = “neither satisfied nor dissatisfied” or “moderate”, 4 = “moderately satisfied” or “high”, and 5 = “completely satisfied” or “extremely high”. 

The disability status was assessed by one question (“What kinds of limitations or participation restrictions in daily life or during work, due to your health problem, do you have?”) with three responses: no disability, a non-work-related disability, or a work-related disability [6,12].

The presenteeism score of the Work Productivity and Activity Impairment Questionnaire: General Health (WPAI:GH) [24,25] was calculated by dividing the degree of sickness-influenced productivity when employees were working by 10 and then multiplying that by 100.

The SPS-6 has six items. Item numbers 2, 5, and 6 are positively worded items: 1 = “strongly disagree”, 2 = “somewhat disagree”, 3 = “uncertain”, 4 = “somewhat agree”, and 5 = “strongly agree”. Three other items have reversed scores. The total score for the SPS-6 ranges from 6 to 30. A higher score indicates greater productivity [6].

### 2.5. Data Analysis

Data were analyzed using R software version 4.2.0 (Vienna, Austria) [26], with the psych, semPaths and lavaan packages. Significance levels were set to 0.05. 

The internal consistency was analyzed by computing Cronbach’s alpha. The value of Cronbach’s alpha had to be at least 0.70 to have acceptable internal consistency reliability [27]. There was a presence of a ceiling or floor effect if >15% of participants received the highest or lowest total SPS-6 scores, respectively [28].

The content validity was evaluated by content validity indices at the item level (I-CVI) and the scale level (S-CVI) [20], the modified Kappa (k*) statistic [29], and the expert panel as follows:

- The I-CVI was computed by dividing the number of experts who evaluated an item as three (quite relevant or minor revisions to be clear) and four (highly relevant or clear) by the total number of evaluators [30]. Because there were fewer than six experts, the I-CVI in our study was 1.00 [31].
- The S-CVI was calculated by averaging I-CVI values [29].
- To adjust for the chance agreement of experts [29], the modified Kappa (k*) statistic was calculated following a formula recommended by Polit et al. [31,32] and interpreted as recommended by Fleiss et al.: excellent (>0.74), good (0.60–0.74), and fair (0.40–0.59) [31,33].
- Through email, we asked the first expert panel whether the translated version was easy to comprehend and would it be clear for all Taiwanese populations with five responses from 1 (“strongly disagree”) to 5 (“strongly agree”).

The structural validity was examined by an exploratory factorial analysis (EFA) and confirmatory factor analysis (CFA).

- Before conducting the EFA, we used the Kaiser–Mayer–Olkin (KMO) test to assess the sampling adequacy. A KMO index ≥0.50 is an acceptable value [34], and Bartlett’s test of sphericity with a significant statistical test (*p* < 0.05) [35] is desirable. The EFA with principal component analysis extraction and Varimax rotation methods was applied to test the dimensions of the CSPS-6. Items with a factor loading of >0.40 were set. Two methods we used to define the number of factors retained were the Kaiser criterion of eigenvalues (eigenvalue >1 rule) [36,37] and a scree plot. Factors to be retained were determined by the eigenvalues above and to the left of the straight line through the smallest eigenvalues on the scree plot [38,39].
- A CFA was performed to confirm the structural validity of the CSPS-6. With a sample size N of ≤250, Hu and Bender (1999) recommended a two-index presentation strategy using the combination rule of fit indexes to conclude the model fit [40]. Therefore, we reported a combination of the comparative fit index (CFI) (or Tucker–Lewis Index (TLI)) with the standardized root-mean-squared residual (SRMR) for model evaluation. Cutoff values for these indices to determine a good model fit were a CFI of ≥0.95 (or a TLI of ≥0.95) and an SRMR of ≤0.08 [40]. The factor loading of an item was interpreted as excellent, very good, good, fair, and poor with loadings exceeding 0.71, 0.63, 0.55, 0.45, and 0.32, respectively [41,42].

Due to skewed data of productivity, Spearman correlation coefficients (r_s_) were used to test the correlation between the SF-36 and the CSPS-6 (Bonferroni adjusted *p*-value < 0.006), the WPAI:GH, job stress, and job satisfaction. According to Cohen’s classification, we interpreted Spearman’s coefficient values of >0.5, 0.3–0.5, and <0.3 as being strong, moderate, and weak, respectively [43]. An analysis of variance (ANOVA) was used to test the relationship between disability status and the CSPS-6. 

## 3. Results

Demographic characteristics are described in Table 1. The mean CSPS-6 score (standard deviation) was 24.11 (5.42). A ceiling effect (maximum score of 30) was present (29%, *n* = 57) for CSPS-6. Only two employees (1%) got the minimum score of 6; therefore, there was no floor effect. The mean job stress score (standard deviation) was 2.55 (1.02). The mean job satisfaction score (standard deviation) was 3.24 (0.92). The mean WPAI:GH presenteeism score (standard deviation) was 24.85 (28.72).

### 3.1. Internal Consistency

Cronbach’s alpha values were 0.98 (for factor 1), 0.83 (for factor 2), and 0.74 (for the whole scale).

### 3.2. Content Validity

In terms of clarity and relevance as evaluated by four experts, all the I-CVI, S-CVI, and Kappa k* values that were equal to 1 presented total agreement of all experts with the probability of a chance occurrence among experts of 0.0625. These results proved that the content validity of the CSPS-6 was excellent.

The original developer of this scale and two native English-speaking researchers strongly agreed that the backward translation had the same content equivalence as the original version. Most experts agreed that the CSPS-6 had translation equivalence with the original English version, and that it was easy to comprehend and would be clear for all Taiwanese populations (one-fourth of experts strongly agreed, two-fourths of experts agreed, and one-fourth had a neutral opinion). 

### 3.3. Structural Validity (Construct Validity)

**Figure 1:**
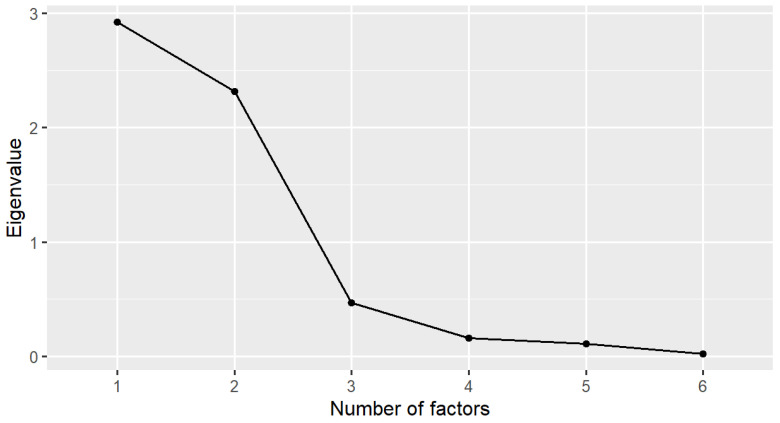
Scree plot of the Chinese version of the Stanford Presenteeism Scale (CSPS-6) in the exploratory factor analysis (EFA).

Because the KMO value was 0.65 and Bartlett’s test of sphericity was significant (χ^2^ = 1287.3, *p* < 0.001, degrees of freedom = 15), it was appropriate to use the EFA. Results of the EFA in Table 2 and Figure 1 suggest that the CSPS-6 had two factors, labeled “completing work” and “avoiding distraction” in the original English version, with eigenvalues >1 that explained 87.4% of the total variance of the responses. 

**Figure 2:**
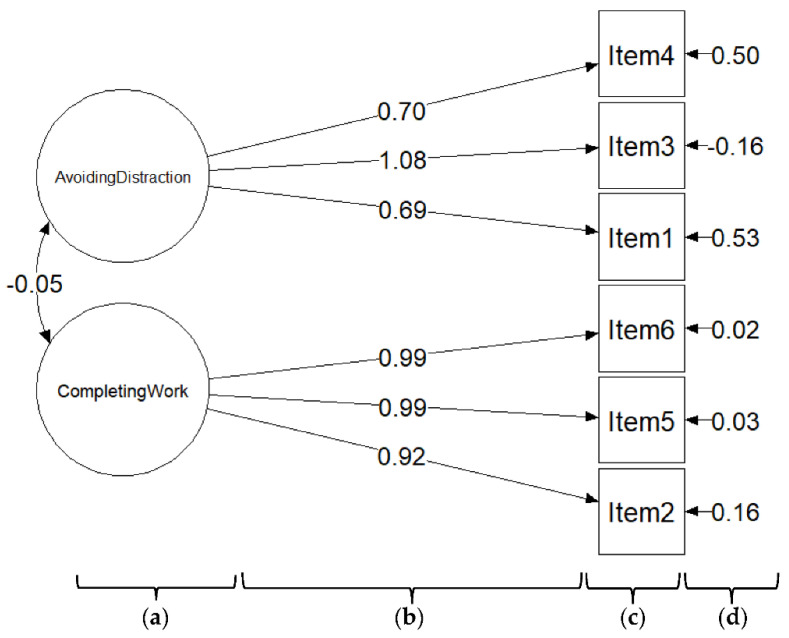
The two-factor model of the CSPS-6 according to a confirmatory factor analysis. (**a**) CSPS-6 factors; (**b**) standardized factor loading; (**c**) CSPS-6 items; (**d**) error variance.

Figure 2 presents the CFA of the CSPS-6 with two factors. The fit indices of the model evaluation were CFI = 0.976, TLI = 0.956, and SRMR = 0.075, indicating model fit. The association between the two factors was −0.05. The standardized factor loadings were in the range of 0.69–1.08 (*p* < 0.05), indicating that the magnitude of the relationships of items to the factor was good, and the CSPS-6 had good construct validity.

### 3.4. Hypothesis Testing (Construct Validity)

The CSPS-6 score was positively correlated with eight outcomes of health status (SF-36) (r_s_ = 0.28–0.52, *p* < 0.0001) (Table 3). Correlations were found between job satisfaction (r_s_ = 0.41, *p* < 0.0001) and job stress (r_s_ = −0.22, *p* = 0.002) with presenteeism. No correlations were found between mean CSPS-6 scores among the three disability groups (*p* = 0.19, F-value = 1.67, degrees of freedom = 2).

### 3.5. Concurrent Validity (Criterion Validity)

The CSPS-6 score was moderately associated with the WPAI:GH presenteeism score (r_s_ = −0.46, *p* < 0.0001).

## 4. Discussion

Our findings show that the CSPS-6 is a valid and reliable questionnaire that might be useful to assess employee health and productivity. Overall, this instrument showed an acceptable internal consistency. Results of the expert panels indicated that the CSPS-6 was clear and relevant to the concept of presenteeism (reduced productivity at work due to illness), and that the meanings of the CSPS-6 and the original English version were similar. This provides proof of the good content validity of the CSPS-6. CSPS-6 was associated with eight dimensions of the health status (SF-36), job stress, and job satisfaction (except the disability status), suggesting that the CSPS-6 had acceptable construct validity. The CSPS-6 score was associated with the WPAI:GH presenteeism score, indicating the acceptable concurrent validity of this scale.

The CSPS-6 showed acceptable internal consistency. This result seems to be consistent with other research which found translated versions of the SPS-6 had good internal consistency (Cronbach’s alpha ≥ 0.8) [6,10,11,12] or acceptable internal consistency [13,14]. The EFA and CFA findings support the structural validity of the CSPS-6, which reflects two dimensions: avoiding distractions (work processes) and completing work (work outcomes). A comparison of the findings with those of other studies [10,11,12,13,14,15,17] confirms that the CSPS-6 has two underlying dimensions, in line with the original English version [6]. Koopman et al. [6] and Hutting et al. [12] proved that SPS-6 scores were associated with the disability status. This differs from findings presented here because the mean age of our respondents (34.08 years old) was less than that of Koopman et al.’s participants (46.5 years old) and Hutting et al.’s participants (46.2 years old).

Our study confirmed the association between health status outcomes (SF-36) and CSPS-6 scores. This finding was also reported by Frauendorf et al. [10]. The reason for this might be that health problems, especially pain, discourage employees from concentrating on their work, which causes presenteeism [44]. In accordance with previous validation studies [6,12], this study also demonstrated that CSPS-6 scores were associated with job satisfaction and job stress. This finding is in agreement with Keramat’s longitudinal study findings which showed that job satisfaction was correlated with presenteeism [45]. The relationship between job stress and presenteeism may partly be explained by job stress, yielding negative emotions and go-slow behaviors that contribute to presenteeism [46]. In addition, when employees are sick but still go to work, stressors might make them have a higher likelihood of errors which affect their work performance [47]. Several limitations might have influenced the results we obtained. First, our participants were employees in technology companies, which might not be generalizable to all Taiwanese employees. Second, this study was cross-sectional research; thus, we were unable to conclude causal relationships between the health status and reduced productivity at work due to illness. Further research is required to assess measurement properties of the CSPS-6 in other working populations and the long-term effects of health problems on presenteeism.

Presenteeism is a key public health problem because it reduces the quality of work output in employees and causes hidden indirect costs for organizations. Our study proved that the CSPS-6 has acceptable reliability and validity to measure productivity loss with less bias because of the lack of valid instruments. Our research can be a basis for future studies on assessing of treatment interventions on employee productivity and health in clinical settings or the effectiveness of health-promotion programs in the workplace to reduce productivity losses among employees.

## 5. Conclusions

In conclusion, the CSPS-6 has acceptable reliability and validity to measure reduced productivity at work due to illness of Taiwanese employees at technology companies. Further research should be undertaken to validate the CSPS-6 in other working populations and assess the long-term effects of health problems on reduced productivity.

## Figures and Tables

**Table 1:** Demographic characteristics (N = 196).

Characteristic	*n* (%)
Age ^+^	34.08 (7.55)
Gender Male	158 (79.6)
Educational attainment	
High school	75 (38.3)
Undergraduate	91 (46.4)
Postgraduate	30 (15.3)
Marital status	
Married/cohabiting	86 (43.9)
Separated/divorced/widowed	5 (2.5)
Never married	105 (53.6)
Type of job	
Manager, professional	26 (13.2)
Technologist	109 (55.6)
Office job, service	61 (31.2)
Disability status	
Work-related disability	135 (68.9)
Non-work-related disability	6 (3.1)
No disability	55 (28.0)

^+^ Mean (standard deviation).

**Table 2:** Factor loadings of the CSPS-6 (principal components analysis extraction and Varimax rotation methods).

Item	Factor 1 (Completing Work)	Factor 2 (Avoiding Distraction)
Item 1		0.845
Item 2	0.959	
Item 3		0.951
Item 4		0.843
Item 5	0.981	
Item 6	0.983	
Total variance explained	48.5%	38.9%
Eigenvalue	2.91	2.33

**Table 3:** Correlations between the CSPS-6 and the health status.

Health Status Outcome	Mean (Standard Deviation)	r_s_
Role—Physical	86.10 (23.91)	r_s_ = 0.31
Physical functioning	94.61 (9.89)	r_s_ = 0.32
Role—Emotional	91.80 (19.56)	r_s_ = 0.28
Pain	84.44 (23.83)	r_s_ = 0.29
Social functioning	84.76 (21.66)	r_s_ = 0.48
Vitality	69.13 (18.16)	r_s_ = 0.52
General health	57.50 (16.66)	r_s_ = 0.47
Mental health	75.51 (16.38)	r_s_ = 0.42

## Data Availability

Not applicable.

## Supplementary Materials

The following supporting information can be downloaded at www.mdpi.com/article/10.3390/healthcare10112202/s1: The original English version and the traditional Chinese version of the Stanford Presenteeism Scale [6].
