# Supplementary material for: Psychometric Properties of the Traditional Chinese Version of the Stanford Presenteeism Scale (SPS-6) among Taiwanese Employees in Technology Companies
Source: Healthcare (Basel). 2022 Nov 2;10(11):2202. doi: 10.3390/healthcare10112202 (PMC9690533; doi:10.3390/healthcare10112202)
Supplement: Supplementary file 1 [file healthcare-10-02202-s001.zip › healthcare-1971946-SI.pdf]

The original English version of Stanford Presenteeism Scale (SPS-6) [6]

Directions: Below we would like you to describe your work experiences in the **past month**. These experiences may be affected by many environmental as well as personal factors and may change from time to time. For each of the following statements, please circle one of the following responses to show your agreement or disagreement with this statement in describing *your* work experiences in the past month.

Please use the following scale:

Circle:

1 if you strongly disagree with the statement
2 if you somewhat disagree with the statement
3 if you are uncertain about your agreement with the statement
4 if you somewhat agree with the statement
5 if you strongly agree with the statement

**Statement**	**Your work experience in the past month:**				
1. Because of my (health problem),* the stresses of my job were much harder to handle.	1	2	3	4	5
2. Despite having my (health problem),* I was able to finish hard tasks in my work.	1	2	3	4	5
3. My (health problem)* distracted me from taking pleasure in my work.	1	2	3	4	5
4. I felt hopeless about finishing certain work tasks, due to my (health problem).*	1	2	3	4	5
5. At work, I was able to focus on achieving my goals despite my (health problem).*	1	2	3	4	5
6. Despite having my (health problem),* I felt energetic enough to complete all my work.	1	2	3	4	5

*** Note that the words “back pain,” “cardiovascular problem,” “illness,” “stomach problem,” or other similar descriptors can be substituted for the words “health problem” in any of these items.**

The Traditional Chinese version of Stanford Presenteeism Scale (CSPS-6) [6]

說明:請您在下節描述在**過去一個月**中的工作經歷。這些經歷可能會受到許多環境以及個人因素的影響並因此經常有所改變。對於以下每一項陳述，請在相關回答中圈出一項，以表示您同意或不同意這項陳述所描述之過去一個月的工作經歷。

請使用以下量表:

圈選:

1 如果您強烈不同意該描述，則圈選1。

2 如果你對該描述有些不同意的話，則圈選2。

3 如果您不確定您是否同意該描述，則圈選3。

4 如果你對該描述有一定程度的認同，則圈選4。

5 如果您非常同意該描述，則圈選5。

**描述**	**您在過去一個月中的工作經歷:**				
1. 因為我的(健康問題)，*工作壓力變得更加難以處理。	1	2	3	4	5
2. 儘管因為我有(健康問題)，*我在工作中還是能完成困難的任務。	1	2	3	4	5
3. 我的(健康問題)*分散了我的注意力，使我無法享受工作的樂趣。	1	2	3	4	5
4. 由於我的(健康問題)，*我對完成某些工作任務感到無望。	1	2	3	4	5
5. 儘管我有(健康問題)，*在工作中我還是能夠專注於實現我的目標。	1	2	3	4	5
6. 儘管我有(健康問題)，*我還是覺得有足夠的精力去完成所有的工作。	1	2	3	4	5

* 請注意，"背痛"、"心血管問題"、"疾病"、"胃病 "或其他類似的描述用詞可以用以代替上述描述中的 "健康問題"。
